# Phylogenetic Relationships, Character Evolution, and Two New Species in *Hemiboea* (Didymocarpoideae, Trichosporeae)

**DOI:** 10.1002/ece3.72330

**Published:** 2025-10-14

**Authors:** Ping Liang, Xiao Wang, Fang Wen, Xin‐Xiang Bai, Jun Mu

**Affiliations:** ^1^ Forestry College Guizhou University Guiyang China; ^2^ Guizhou Xishui National Nature Reserve Management Bureau Xishui Guizhou China; ^3^ Guangxi Institute of Botany, Guangxi Zhuang Autonomous Region and Chinese Academy of Sciences Guangxi Key Laboratory of Plant Conservation and Restoration Ecology in Karst Terrain Guilin Guangxi China; ^4^ Gesneriad Committee of China Wild Plant Conservation Association National Gesneriaceae Germplasm Resources Bank of GXIB, Gesneriad Conservation Center of China Guilin Guangxi China

**Keywords:** character evolution, China, Gesneriaceae, phylogeny, taxonomy

## Abstract

Two new species, *Hemiboea xishuiensis* X.X. Bai and *Hemiboea cehengensis* X.X. Bai, from Guizhou, China, are described here. We performed a molecular phylogenetic analysis of the two new species and 28 taxa within *Hemiboea* based on ITS and *trnL‐F*, which shows that the phylogenetic tree of *Hemiboea* forms three major clades: *H. fangii* Chun ex Z.Yu Li occupies one clade exclusively (Clade I), *H. xishuiensis*, *H. cehengensis*, *H. parvibracteata* W.T. Wang & Z.Yu Li, *H. ovalifolia* (W.T. Wang) A. Weber & Mich. Möller, and *H. kaiyangensis* T. Peng & S.Z. He constitute a highly supported clade (Clade II), and the remaining taxa are clustered into the third clade (Clade III). Furthermore, this study traced the evolution of eight important morphological characters in *Hemiboea* and determined their ancestral states. The results show that the involucre is not early deciduous, the calyx is 5‐sect from the base, the corolla is hairy outside, there are two protuberances and trichomes on the inner abaxial surface of the corolla, and there is a hairy ring above the base on the inner face of the corolla, the ovary is glabrous, and these characters may be the ancestral states of *Hemiboea*; *H. fangii* is the most primitive species of *Hemiboea* found to date; whether the involucre is early deciduous and whether there are two protuberances on the inner ventral surface of the corolla may be the key characters for the classification of sections under *Hemiboea*. This study not only enriches the species diversity of *Hemiboea* in China but also provides a new inspiration for the infrageneric classification of *Hemiboea*.

## Graphical abstract

This paper describes two new species of Hemiboea based on molecular and morphological evidence. Furthermore, we trace the evolution of eight important morphological characters in Hemiboea, which indicates whether the involucre is early deciduous and whether there are two protuberances on the inner ventral surface of the corolla, which may be the key characters for the classification of sections under Hemiboea.

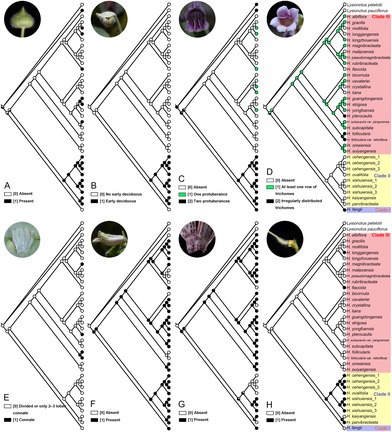

## 1 Introduction

*Hemiboea* C.B. Clarke is a small genus of perennial herbs in Gesneriaceae. Most taxa of the genus occur in southern China, with a few taxa in northern Vietnam, northern Laos, and southern Japan (Wang et al. 1990; Souvannakhoummane et al. 2021), mainly growing on limestone substrates (Wei et al. 2004). The genus can be recognized by the bracts connate into spherical, ovoid, or trigonous involucres, the ovary 2‐loculed with only one locule fertile, and the old capsule boat‐shaped, opening only along the upper side (Wang et al. 1998; Huang et al. 2024; Möller et al. 2017). The genus was originally described by the British scholar C.B. Clarke (1888) using *H. follicularis* C.B. Clarke as the type species. He divided the then three taxa into two infrageneric sections based on five morphological characters: inflorescence, calyx, corolla, disc, and anthers. Sect. *Sympodiales* contained one taxon: *H. follicularis* , and sect. *Subcapitate* contained two taxa: *H. subcapitata* C.B. Clarke and *H. henryi* C.B. Clarke. Later, Z.Y. Li (1987) simplified the taxonomic characters for the infrageneric classification because the five morphological characters Clarke used no longer supported phylogenetic relationships within the genus, and Li divided *Hemiboea* into two sections based on whether the calyx lobes are united or not: sect. *Subcapitatae* Clarke and sect. *Hemiboea* Z.Y. Li. With the development of intensive fieldwork by botanists, a large number of new taxa of *Hemiboea* have been published. As of December 31, 2024, 45 species and 5 varieties of *Hemiboea* have been recorded (Wen et al. 2024). Molecular data have been essential in systematically placing taxa in the correct taxonomic status (Möller et al. 2011; Weber, Middleton, et al. 2011; Weber, Wei, et al. 2011; Huang et al. 2017; Li et al. 2019). Thus, to support new taxa, researchers have increasingly included molecular analyses in studies, such as Huang et al. (2020), Peng et al. (2022), and Huang et al. (2024), which have also challenged existing infrageneric classifications for *Hemiboea*—these studies showed that sect. *Hemiboea* is nested within the sect. *Subcapitatae*. Thus, the infrageneric phylogenetic relationships of this genus still need to be resolved.

In September 2023, we discovered two unknown *Hemiboea* species in Guizhou Province, China. In order to determine their phylogenetic position, we sampled 26 species and 2 varieties within *Hemiboea* and reconstructed the phylogenetic tree of *Hemiboea* using the internal transcribed spacer sequences (ITS) of ribosomal DNA and the molecular data of *trnL* intron‐*trnL‐F* intergenic spacer region of chloroplast DNA (*trnL‐F*). Following this, we combined the results with morphological analyses and confirmed that these two species were previously undescribed. Besides, in the process of this work, we found that the clade in which the two new species are found shares some characters that differ from those of the other two clades. So, in order to test which traits are diagnostic for the three major clades inferred on the basis of molecular data to preliminarily explore the infrageneric phylogenetic relationships of the genus, we conducted an analysis of morphological character evolution in *Hemiboea* plants within a new phylogenetic framework.

## 2 Materials and Methods

### 2.1 Morphological Observations and Description of Two New Species

The plants of two new species were dissected in detail and photographed, and their morphological characters were carefully measured and recorded. To identify the taxa and to ascertain the status of the new taxa, we consulted *Flora of China (vol. 18)* and followed the keys to the species and genera of *Flora of China (vol. 18)* to search for their morphologically allied taxa reported in the past. Then, the two new taxa were compared with original descriptions and specimens of morphologically allied taxa, including about 60 specimens deposited in the herbaria of CSH, E, GNUB, GXMG, GZAC, GZTM, HGCM, IBK, IBSC, JIU, KUN, NAS, PE, SYS, and WUK collected in Guizhou, Guangxi, Hunan, Hubei, and Jiangxi, China. The morphological characters of these two new species were described with reference to the terminology used by Wang et al. (1998) for morphological characterization.

We also observed the pollen morphology of two new species: firstly, we cut off their stamens during the flowering period, dried, and preserved them in silica gel in order to bring them back to the laboratory conveniently. Secondly, we crushed the anthers and made the pollen stick as evenly as possible on the black double‐sided tape, and then vacuum‐coated them. Finally, we scanned, observed, and photographed them with a SU8100 scanning electron microscope. Refer to Luegmayr (1993) for terms describing pollen morphology.

### 2.2 Taxonomic Sampling, DNA Sequencing, and Molecular Analyses

In order to determine the phylogenetic position of the two new species by using ITS and *trnL‐F*, we selected 26 species and 2 varieties as the ingroup, and based on Möller et al. (2011) and Weber, Wei, et al. (2011), two species of *Lysionotus* D. Don: *Lysionotus petelotii* Pellegr. and *Lysionotus pauciflorus* Maxim. were treated as the outgroup. Thus, we sampled a total of 30 taxa, of which sequence data for seven taxa were obtained from sequencing in this study and for 23 taxa were obtained from the GenBank database in the National Center for Biotechnology Information (NCBI, https://www.ncbi.nlm.nih.gov/nuccore) (Table 1). Except for the leaves of *Hemiboea subacaulis* var. *jiangxiensis* Hand.‐Mazz., which were taken from plants introduced for cultivation in Guizhou Provincial Botanical Garden, the leaf materials of the remaining six newly sequenced species were obtained from specimens deposited in the herbarium of Forestry College, Guizhou University (GZAC). The leaf materials of the two new species were collected from the healthy, fresh, and mature leaves in the middle and upper parts of the plants of the type locality, and were dried with silica gel for rapid drying. The molecular methods and protocols for obtaining ITS and *trnL‐F* sequences followed Möller et al. (2009) and Weber, Middleton, et al. (2011).

**TABLE 1:** List of taxa sampled and NCBI accession numbers of sequences used in phylogenetic analyses of *Hemiboea*.

Taxon	Voucher	ITS	*trnL‐F*	References
*Hemiboea albiflora* X.G. Xiang, Z.Y. Guo & Zhao W.Wu	X.X.Bai BXX‐bs‐006 (GZAC)	PV439821	PV453508	This study
*H. bicornuta* (Hayata) Ohwi	Smithsonian Institute [cultivation]	FJ501356	FJ501534	Möller et al. (2011)
*H. cavaleriei* H. Lév.	X.X.Bai BXX‐bs‐020 (GZAC)	PV439818	PV453505	This study
*H. cehengensis* X.X. Bai	—	PV448324–26	PV453512–14	
*H. crystallina* Y.M. Shui & W.H. Chen	SYM B2005‐12 (KUN)	MN334632	—	Li et al. (2019)
*H. fangii* Chun ex Z.Yu Li	M.Möller MMO 08–1284 (E)	HQ632979	HQ632883	Möller et al. (2011)
*H. flaccida* Chun ex Z.Yu Li	YC 1501 (PE)	MN334634	—	Li et al. (2019)
*H. follicularis* C.B. Clarke	Y.G.Wei G03 (IBK)	HQ632983	HQ632885	Möller et al. (2011)
*H. follicularis* var. *retroflexa* Yan Liu & Y.S. Huang	X.X.Bai BXX‐bs‐026 (GZAC)	PV439820	PV453507	This study
*H. gracilis* Franch.	LZY & XXG 20150604009 (PE); Y.Z. Li 11317 (PE)	MN334635	FJ501536	Li et al. (2019); Möller et al. (2011)
*H. guangdongensis* (Z.Y.Li) X.Q. Li & X.G. Xiang	Peiqiong Li s.n. (PE)	MF625025	—	Huang et al. (2017)
*H. kaiyangensis* T. Peng & S.Z. He	Shun‐Zhi He 090819 (HGCM)	JN644335	JN644339	Peng et al. (2022)
*H. liana* Z.P. Huang, Y.B. Lu & B. Pan	—	MW035004	MW090969	Huang et al. (2024)
*H. longzhouensis* W.T. Wang ex Z.Yu Li	M.Möller MMO 07–1127 (E)	HQ632985	HQ632888	Möller et al. (2011)
*H. longgangensis* Z.Yu Li	Y.G.Wei 07–550 (IBK)	HQ632986	HQ632889	
*H. magnibracteata* Y.G. Wei & H.Q. Wen	M.Möller MMO 08–1347 (E)	HQ632984	HQ632887	
*H. malipoensis* Y.H. Tan	JXH 16804 (PE)	MN334639	—	Li et al. (2019)
*H. mollifolia* W.T. Wang	X.X.Bai BXX‐bs‐014 (GZAC)	PV439819	PV453506	This study
*H. ovalifolia* (W.T.Wang) A. Weber & Mich. Möller	N.B.Ming 06–1 (IBK)	HQ632980	HQ632883	Möller et al. (2011)
*H. omeiensis* W.T. Wang	M.Möller MMO 08–1271 (E)	HQ632983	HQ632886	
*H. pterocaulis* (Z.Y.Li) J. Huang, X.G. Xiang & Q. Zhang	J. Huang 16051101 (PE)	KY607398	KY607416	Huang et al. (2017)
*H. parvibracteata* W.T. Wang & Z.Yu Li	X.X.Bai BXX‐bs‐015 (GZAC)	PV439816	PV453503	This study
*H. pseudomagnibracteata* B. Pan & W.H. Wu	Pan B YC2012 (PE)	KY288035	—	Huang et al. (2020)
*H. rubribracteata* Z.Yu Li & Yan Liu	M.Möller MMO 07–1093 (E)	HQ632987	HQ632890	Möller et al. (2011)
*H. strigosa* Chun ex W.T. Wang	HMQ G015 (PE)	MN334647	—	Li et al. (2019)
*H. subacaulis* var. *jiangxiensis* Hand.‐Mazz.	Guizhou Provincial Botanical Garden [cultivation]	PV448327	PV453515	This study
*H. subcapitata* C.B. Clarke	Y.Z.Wang 11306 (PE)	FJ501357	FJ501535	Möller et al. (2011)
*H. suiyangensis* Z.Yu Li, S.W. Li & X.G. Xiang	X.X.Bai BXX‐bs‐017 (GZAC)	PV439817	PV453504	This study
*H. xishuiensis* X.X. Bai	—	PV448321–23	PV453509–11	
*H. yongfuensis* Z.P. Huang & Y.B. Lu	—	MK441665	MK441675	Huang et al. (2020)
Outgroup				
*Lysionotus pauciflorus* Maxim.	M.Möller MMO 01–101 (E, WU)	FJ501331	FJ501497	Möller et al. (2009)
*L. petelotii* Pellegr.	M.Möller MMO 01–100/4 (E); M.Möller MMO 01–100 (E, WU)	HQ632974	FJ501496	Möller et al. (2009, 2011)

The ITS and *trnL‐F* sequences of the taxa used were aligned separately using MEGA v.11.0.13 software (Tamura et al. 2021), and the missing portions of the sequences at both ends were manually trimmed appropriately after alignment. The optimal partitioning strategy and evolutionary model selection most suitable for the concatenated dataset were determined by PartitionFinder2 (Lanfear et al. 2016) using the corrected Akaike Information Criterion (AICc). Then, phylogenetic trees were constructed using maximum likelihood (ML) methods at IQ‐TREE (Nguyen et al. 2015) and Bayesian inference (BI) at MrBayes v.3.2.7 (Ronquist et al. 2012). The Bayesian inference was run in two parallel runs with four Markov chain Monte Carlo (MCMC) chains for 2,000,000 generations, sampled once every 1000 generations, and the convergence of the two independent analyses was determined by the standard deviation of the splitting frequency of less than 0.01. Posterior probabilities were determined from the posterior distribution after discarding the first 25% trees of each run as burn‐in. The maximum likelihood methods used 1000 bootstrap analyses to verify the reliability of each branch. The phylogenetic trees and branch support values were visualized using iTOL v.6 (Letunic and Bork 2024).

### 2.3 Reconstructing the Ancestral State of Some Selected Morphological Characters

To test which traits are diagnostic for primary clades, trace the evolution of these morphological features and determine their ancestral states. Ancestral character state analysis was performed using the software Mesquite v.3.81 (Maddison and Maddison 2023) under the phylogenetic framework of *Hemiboea* derived from the Bayesian inference method of the concatenated datasets (combined ITS and *trnL‐F* sequence matrices) with the Likelihood Ancestral States reconstruction method, and the Markov k‐state 1 (Mk1) parameter model was chosen as the probability model used in this analysis. Eight morphological characters were selected for analysis in this study, including five characters that have been frequently used in past classifications of *Hemiboea* (Wang et al. 1998), as well as three important taxonomic characters that have not traditionally been used: involucre developmental characters, protuberances, and trichomes on the inner ventral surface of the corolla. These eight characters were chosen from all the characters scored because they are stable qualitative characters and may represent important adaptations in the evolution of the species in *Hemiboea*. All morphological characters were derived from the original literature and confirmed in field surveys, querying the type specimens and the Plant Picture Bank of China (PPBC; http://ppbc.iplant.cn/). Characters were unordered and equally weighted, and morphological characters and their status were coded as follows: (A) outside of involucre: glabrous [0], hairy [1]; (B) developmental characters of involucre: early deciduous [0], not early deciduous [1]; (C) corolla structure: inner abaxial surface without protuberance [0], inner abaxial surface with two protuberances [1]; inner abaxial surface with one protuberance [2]; (D) corolla ventral inner surface: without trichomes [0], with trichomes arranged in at least one row [1], irregularly distributed trichomes [2]; (E) calyx connate or divided: divided or 2–3 lobes united only [0], connate [1]; (F) outside of corolla: glabrous [0], hairy [1]; (G) above inner base of corolla: glabrous [0], with a hairy ring [1]; (H) ovary: glabrous [0], hairy [1].

## 3 Results

### 3.1 Morphological Analysis of Two New Species

*Hemiboea xishuiensis* sp. nov. is similar to *H. parvibracteata* W.T. Wang & Z.Yu Li in its small involucre falling off from the base before flowering (known as early deciduous), the purple speckled pistil, and the hairy ovary, but it can be distinguished by the color and shape of the corolla, and whether a hairy ring is present above the inner base of the corolla. After *H. flaccida* Chun ex Z.Yu Li, *H. parvibracteata* , *H. ovalifolia* (W.T. Wang) A. Weber & Mich. Möller, and *H. crystallina* Y. M. Shui & W. H. Chen, *H. cehengensis* sp. nov. is the fifth species of *Hemiboea* without a hairy ring above the inner base of the corolla. Morphologically, *H. cehengensis* is the most similar to *H. ovalifolia* in terms of the hairiness of the entire plant, the long straight peduncle, the involucre being early deciduous, and the yellowish color of the corolla, from which it differs by green stem, corolla without purple spots and stripes, the narrowly ovate or broadly ovate anthers, and the position of hairs on the pistil.

### 3.2 Phylogenetic Analysis

**FIGURE 1:**
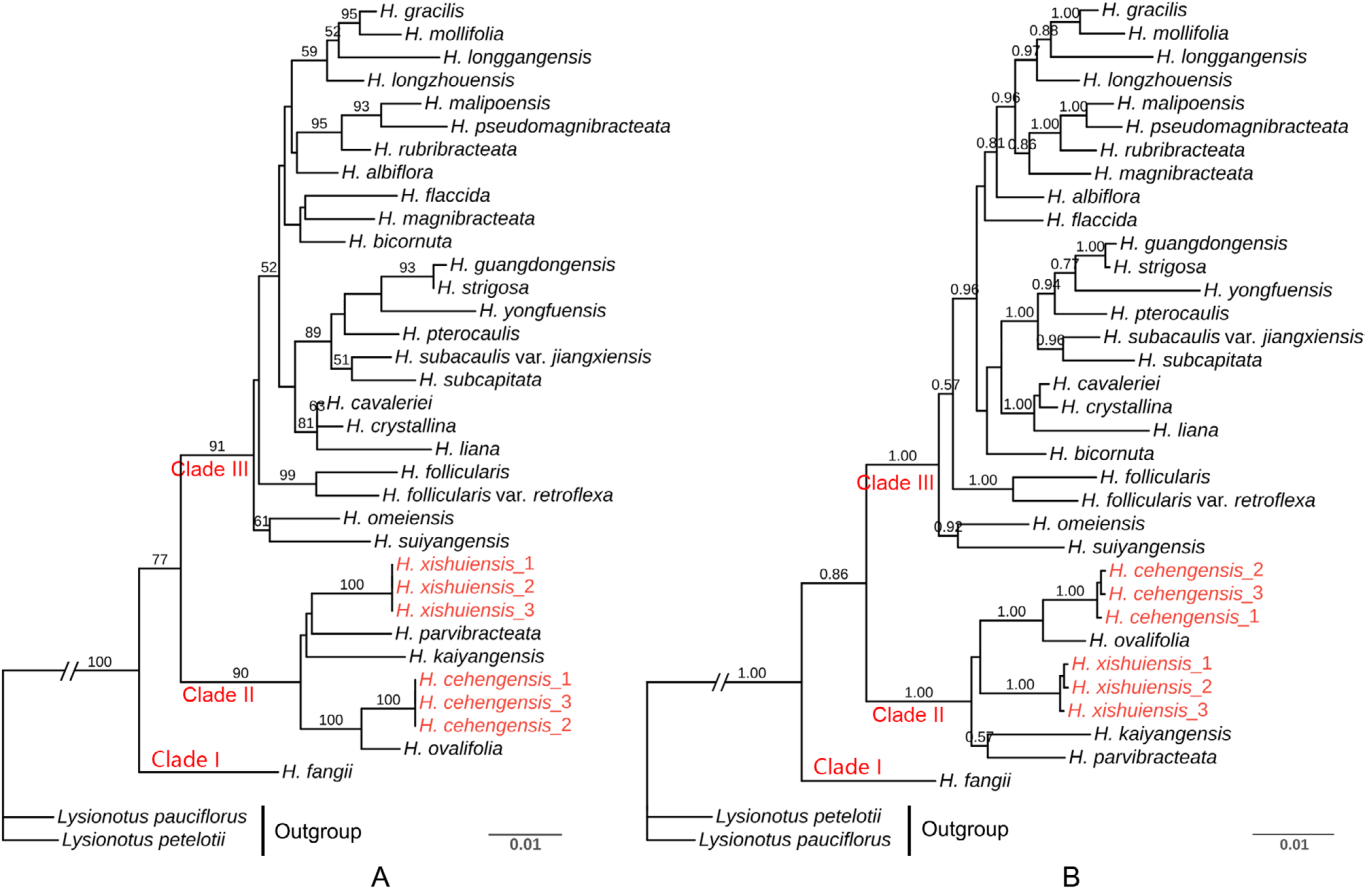
Reconstructed phylogenetic trees of *Hemiboea*. (A) Maximum likelihood (ML) tree based on concatenated ITS and *trnL‐F*, ML bootstrap (BS) values are presented above branches, only showing BS values ≥ 50%; (B) Bayesian inference (BI) tree based on concatenated ITS and *trnL‐F*, BI posterior probabilities (PP) are presented above branches, only showing posterior probabilities ≥ 0.50.

The lengths of the aligned matrix of ITS and *trnL‐F* are 429 bp and 766 bp, respectively. The concatenated matrix is 1195 bp long, including 119 variable sites and 162 parsimony informative sites. Phylogenetic analyses show that the phylogenetic tree topologies constructed based on the combined datasets using both maximum likelihood and Bayesian inference methods are not identical, but *Hemiboea* forms three major clades (Clade I, II, and III) in both the Bayesian inference tree and the maximum likelihood tree (Figure 1). *H. fangii* Chun ex Z.Yu Li is exclusive to Clade I and is diverged from the genus initially. *H. xishuiensis*, *H. cehengensis*, *H. parvibracteata* , *H. ovalifolia* , and *H. kaiyangensis* form Clade II with strong support (PP = 1.00, BS = 90%). Among them, *H. cehengensis* is closely related to *H. ovalifolia*; they form a sister taxon (PP = 1.00, BS = 100%), while the sister taxa of *H. xishuiensis* are poorly revealed. The others are clustered into Clade III with strong support (PP = 1.00, BS = 91%). Clade II and Clade III are sister clades with moderate support (PP = 0.86, BS = 77%), and Clade I is the sister clade to the clade composed of Clade II and Clade III.

### 3.3 Ancestral Character State Reconstruction

**FIGURE 2:**
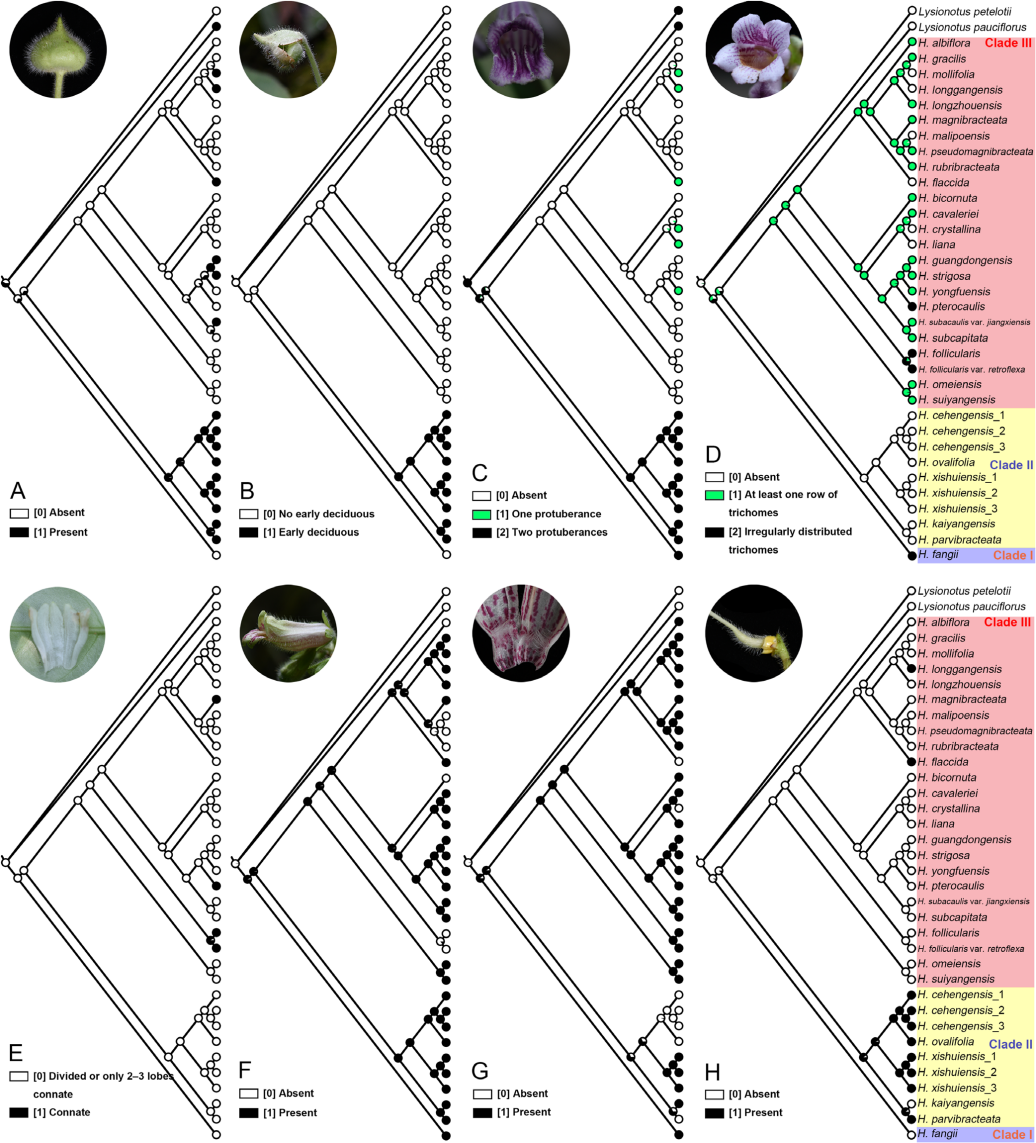
Reconstruction of ancestral states for eight morphological characters using Mesquite. (A) Presence or absence of hairs outside involucre; (B) Growth characters of involucre; (C) Number of protuberances on inner abaxial surface of corolla; (D) Distribution of trichomes on inner abaxial surface of corolla; (E) Fusion or division of sepals; (F) Presence or absence of hairs outside corolla; (G) Presence or absence of hairy ring; (H) Presence or absence of hairs outside ovary.

The results of reconstructing the ancestral state in Mesquite are shown in Figure 2. The involucre is not early deciduous, calyx divided, corolla outside hairy, inner abaxial surface with disorganized trichomes and two protuberances, a hairy ring present on the corolla inner face above the base, and ovary glabrous may be the ancestral state of the last common ancestor of *Hemiboea*. This result also suggests that *H. fangii* is the most primitive species of *Hemiboea* that is currently found, as it conforms to all of the ancestral trait states we have reconstructed. As can be seen in Figure 2, the taxa of clade II share the following morphological characters: hairy and early deciduous involucre, calyx 5‐lobed to the base, corolla hairy outside, inner abaxial surface without trichomes but with two protuberances. The taxa of clade II share two characters that are opposite to those of clade III: the involucre is not early deciduous, and the corolla lacks two protuberances. This suggests that developmental characters of involucre and corolla ventral morphology are diagnostically important in distinguishing the three main clades, clade I, clade II, and clade III.

Evolutionary analysis shows that the character state “involucre early deciduous” occurred only once independently, probably existed before the divergence of clade II and III (Figure 2B), and then, during evolution, this new character was genetically retained in clade II and never originated independently in clade III. The ancestral state that corolla had two protuberances on the inner ventral surface was inherited in clade II and disappeared independently at the formation of clade III (Figure 2C), replaced by regularly arranged trichomes (Figure 2D). The presence of one protuberance but absence of trichomes, and absence of both trichomes and protuberances on the inner ventral surface of the corolla had evolved independently many times in clade III, indicating parallel or convergent evolution. Clade II may still have some taxa with trichomes present on the inner abaxial surface of the corolla at the time of its formation (Figure 2D), and this character was lost in subsequent evolution, which may mean that this character is no longer important during the environmental response of clade II (Ortiz‐Medrano et al. 2016). Compared to clade II, the taxa of clade III are rich and widely distributed, which may imply that such a trait combination “involucre + neatly arranged trichomes on the inner ventral surface of corolla” increases ecological opportunities for clade III and facilitates speciation (Simpson 1944; Gavrilets and Losos 2009; Yoder et al. 2010).

## 4 Discussion

Molecular data has been essential in systematically placing a new species in the correct genus. Molecular data comes principally from three different genomes: mitochondrial, chloroplast, and nuclear. The selection of suitable candidate markers heavily depends on the taxonomic range studied (Möller et al. 2024). At the species level, for example, most often the nuclear ribosomal internal transcribed spacers (ITS) and chloroplast intron/spacers (e.g., *atpB‐rbcL*, *rpl16*, *trnL‐F*, *trnH‐psbA*) are used (e.g., Möller et al. 2009; Li et al. 2019; Liu et al. 2025). Congruence of the distribution of morphological characters with the clade structure of molecular phylogenetic trees is seen as a critical step in the delimitation of taxa, including new ones (Möller et al. 2024). Cui (2023) constructed a phylogenetic tree of *Hemiboea* based on the plastid genome, and the results showed that *Hemiboea* is a well‐supported monophyletic group, but the relationships among the major clades are not well resolved, as the combination of morphological data could not reveal the correlation of these clades, which may be related to incomplete lineage sorting, hybridization/introgression, and horizontal gene transfer. In addition to this, the phylogenetic tree construction of the plastid genome as a single locus may be subject to erroneous inferences of phylogenetic relationships from conflicting loci (Zhang, Wang, et al. 2020; Zhang, Sun, et al. 2020; Bjornson et al. 2024). Our study, which used ITS combined with *trnL‐F* to reconstruct the phylogenetic tree of *Hemiboea* and combined with morphological analyses, supports the status of the new taxa; in addition, the three major clades formed in the phylogenetic tree are also supported by morphological characters. This implies that the molecular approach of ITS combined with *trnL‐F* is effective for the study of infrageneric classification in *Hemiboea*.

Further analysis of morphological characters based on molecular phylogeny will help us understand the origin, diversification, and evolutionary history of morphology. Our character evolutionary analyses indicate the possible phylogenetic significance of involucral developmental characters and corolla ventral characters for the reconstruction of the infrageneric classification system, especially the rank of section in *Hemiboea*, because these characters are clearly and steadily differentiated among the three clades. This result will provide a new inspiration for the infrageneric classification of *Hemiboea*; moreover, this discovery draws our attention to the beautiful corolla and the distinctive bracts of *Hemiboea* plants. In the past 150 years, great attention has been paid to plant bracts. Bracts can protect the floral organs from strong ultraviolet radiation, and as specialized leaves, green bracts have the capacity for photosynthesis (Song et al. 2024). In *Hemiboea*, bracts remain on plants for different lengths of time: bracts of clade II fall off from the base before flowering, while bracts of clade I and clade III can remain throughout the flowering period. Bracts that abscise early may mean less energy expenditure, thereby focusing resources on flower development or reducing interference with pollination. There are currently no studies on the evolutionary mechanisms and drivers of early deciduous bracts in the genus. Floral traits are often associated with pollination mechanisms, and the differentiation of corolla ventral characters in clade II and clade III may imply a shift in pollinators or pollination strategies, but the exact evolutionary drivers are unknown. Whether there is a link between the difference in bract retention time between clade II and clade III and the corolla ventral morphology requires further investigation.

## 5 Taxonomic Treatment

### 5.1 *Hemiboea xishuiensis* X.X. Bai, sp. nov*.*

**FIGURE 3:**
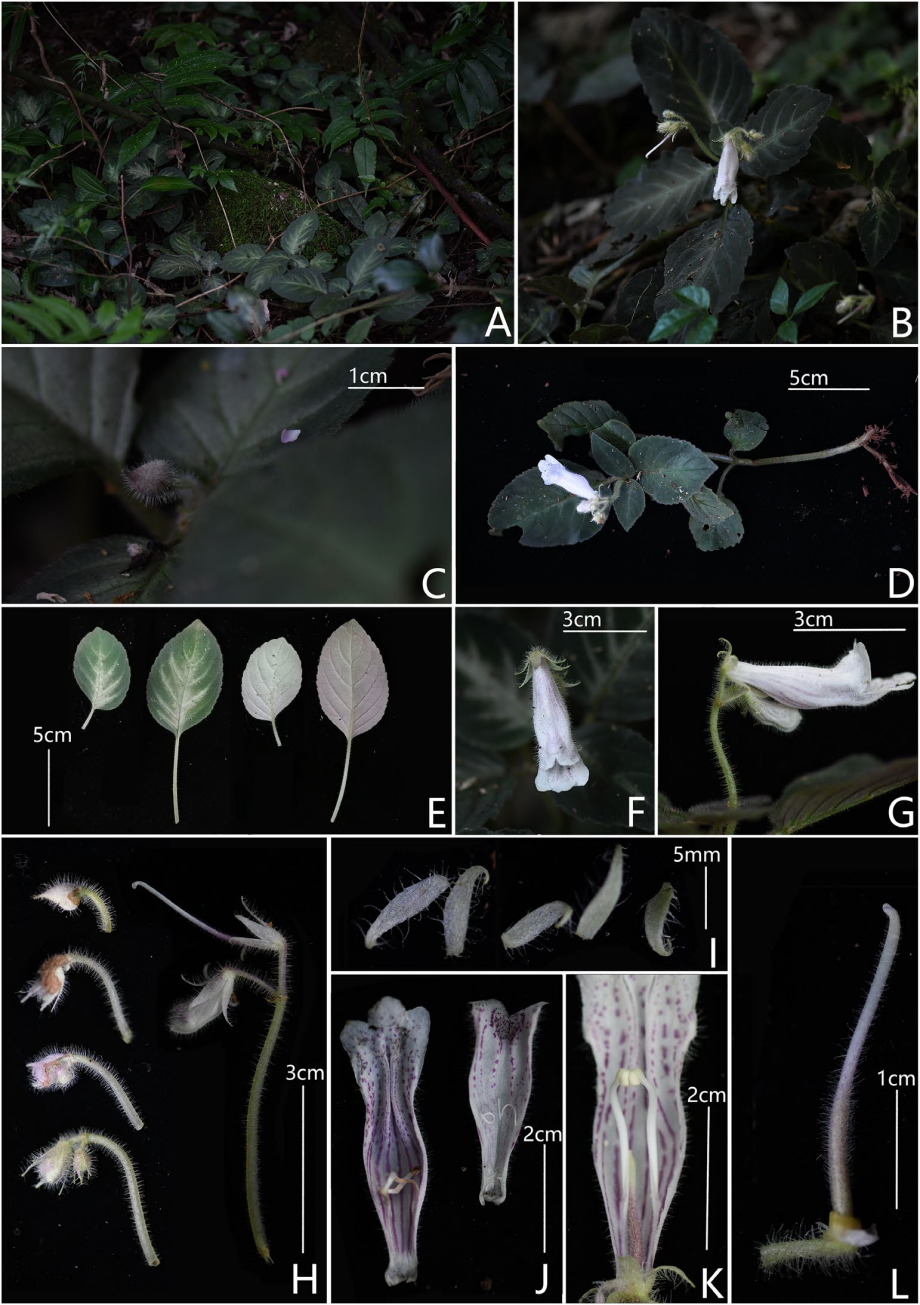
*Hemiboea xishuiensis* sp. nov. (A) Habitat; (B) Flowering individual; (C) Involucre, early deciduous before flower opening; (D) Plant; (E) The adaxial surface of leaf blades and the abaxial surface of leaf blades; (F) Side view of corolla; (G) Lateral view of corolla; (H) Cymes, showing growth changes; (I) Opened calyx; (J) Opened corolla; (K) Stamens; (L) Disc and pistil.

Type: CHINA. Guizhou: Xishui, Linjiang Village, alt. 1160 m, 28°8′46.6″ N, 105°53′27.6″ E, 1 September 2023, voucher *X.X. Bai Bxx‐bs‐031* (holotype, GZAC!; isotype, GZAC!) (Figure 3).

### 5.2 Diagnosis

This species is similar to *Hemiboea parvibracteata* , but differs from *H. parvibracteata* in that its leaf blade is oblong to ovoid (vs. obovate or obovate‐lanceolate), with serrate or repand‐crenate margins (vs. entire), shorter pedicel (0.5–1.9 cm vs. 3–5 cm), corolla is white (vs. purple), and the corolla base is gradually narrowed and has a hairy ring inside, which are absent in *H. parvibracteata* . The detailed morphological comparisons are listed in Table 2.

**TABLE 2:** Morphological comparison of *H. xishuiensis* and *H. parvibracteata* .

Character	*H. xishuiensis*	*H. parvibracteata*
Leaf blade	Oblong to ovoid, with serrate or repand‐crenate margins	Obovate or obovate‐lanceolate, margin entire
Pedicel	0.5–1.9 cm long, densely pubescent with long glandular hairs	3–5 cm long, densely pubescent with glandular‐pubescent
Corolla	White, base gradually narrowed, inside with a hairy ring above the corolla tube base	Purple, base not narrowed, inside without a hairy ring
Filaments of stamens	Sparsely glandular‐pubescent below the middle	Glabrous
Anthers	Suborbicular, completely coherent at the ventral surface	Suborbicular, coherent at the upper part of the ventral surface

### 5.3 Description

Perennial herb. Stems ascending, 9.2–17 cm high, simple or branched, fleshy, green, densely villous. Leaves opposite; petiole 1.4–5.8 cm, densely pubescent; leaf blade oblong to ovoid, 5.4–7.8 × 3.8–5.1 cm, apex acute, base oblique, cuneate to truncate, with serrate or repand‐crenate margins, densely pubescent on both sides, lateral veins 4–7 on each side of midrib. Cymes 1–2, subterminal, 2–8 flowered; peduncle 2.6–4.9 cm long, densely pubescent with long glandular hairs; involucre nearly spheroidal, apex cuspidate, 3–5 mm in diameter, whitish‐green with purple spots, outside densely covered with long glandular hairs, inside glabrous. Pedicel 0.5–1.9 cm long, densely pubescent with long glandular hairs. Calyx 5‐lobed to base, green, lobes linear‐lanceolate, apex cuspidate, 9–11 mm long, 1.5–2.0 mm wide, outside densely covered with long glandular hairs, inside glabrous. Corolla white, with purple spots or stripes inside, 5.0–5.5 cm long, funnelform tube, inflated at middle, narrowed at base, densely glandular‐pubescent outside, inside with a hairy ring adnate to ca. 3 mm above the corolla tube base and with two protuberances on the inner abaxial surface near the mouth; tube 3.5–3.9 cm long, mouth 1.1–1.5 cm in diameter; limb 2‐lipped, adaxial lip 2‐lobed, lobes equal, semiorbicular, 4.2–6.1 mm long, 7.5–9 mm wide; abaxial lip 3‐lobed, central lobe ovate‐triangular, 4–5 mm long, 6.2–7.4 mm wide, lateral lobes oblique triangle, 1.3–1.7 cm long, 4–5.1 mm wide. Stamens 2, adnate to 1.4–1.7 cm above the corolla base; filaments linear, 1.5–2.1 cm long, sparsely glandular‐pubescent below the middle; anthers suborbicular, 1.5–2 mm long, completely connate at the venter surface. Staminodes 3, adnate to 5–15 mm above the corolla base, linear, glabrous, the central one 2–4 mm, the lateral two 6–7 mm. Disk annular, yellow, 1.5–1.9 mm high. Pistil 2.2–2.7 cm long, with purple spots; ovary linear, 9–10 mm long, 1.0–1.8 mm in diameter, densely glandular‐pubescent; style 1.3–1.7 cm long, sparsely glandular‐pubescent, stigma capitate, middle slightly depressed. Capsule unknown.

### 5.4 Pollen Description

The pollen grains of *H. xishuiensis* are oblong, spherical, and trilobate‐rounded in polar view. The pollen grain polar axis length is 24.21–24.43 μm; the equatorial axis length is 17.30–17.32 μm; the germination furrow is 2.12–2.76 μm wide; and the outer wall ornamentation is reticulate, with rough ridges, fewer perforations, and rounded and blunt mesh holes whose size and shape are irregular (See Appendix S1 for images).

### 5.5 Phenology

Flowering period: September and October.

### 5.6 Etymology

The etymology of the specific epithet “xishuiensis” is Latinized from the Chinese Pinyin of the type locality “Xishui” with the suffix “ensis”.

### 5.7 Vernacular Name

Simplified Chinese: 习水半蒴苣苔; Chinese pinyin: xí shuǐ bàn shuò jù tái.

### 5.8 Distribution and Ecology

We focused our survey on Gulin County, Sichuan Province; Xishui County, and Chishui City, Guizhou Province. No additional distribution points for *Hemiboea xishuiensis* were found for the time being, and this species has currently been found at only one distribution point in Xishui County, Guizhou Province, China. It grows in the shaded soil under broad‐leaved evergreen forests in the Danxia landscape and grows together with plants such as *Selliguea lehmannii* (Mett.) Christenh., *Boehmeria clidemioides* var. *diffusa* (Wedd.) Hand.‐Mazz., *Selaginella tamariscina* (P. Beauv.) Spring, and *Pilea pumila* (L.) A. Gray. Although its habitat is located in a national nature reserve and there is no significant threat to the plant and its habitat from human beings, the only population of *Hemiboea xishuiensis* currently discovered has less than 100 mature individuals. Therefore, based on criterion D of IUCN Red List Categories and Criteria (IUCN Standards and Petitions Committee 2024), we propose that the species be considered Endangered (EN).

### 5.9 *Hemiboea cehengensis* X.X. Bai, sp. nov*.*

**FIGURE 4:**
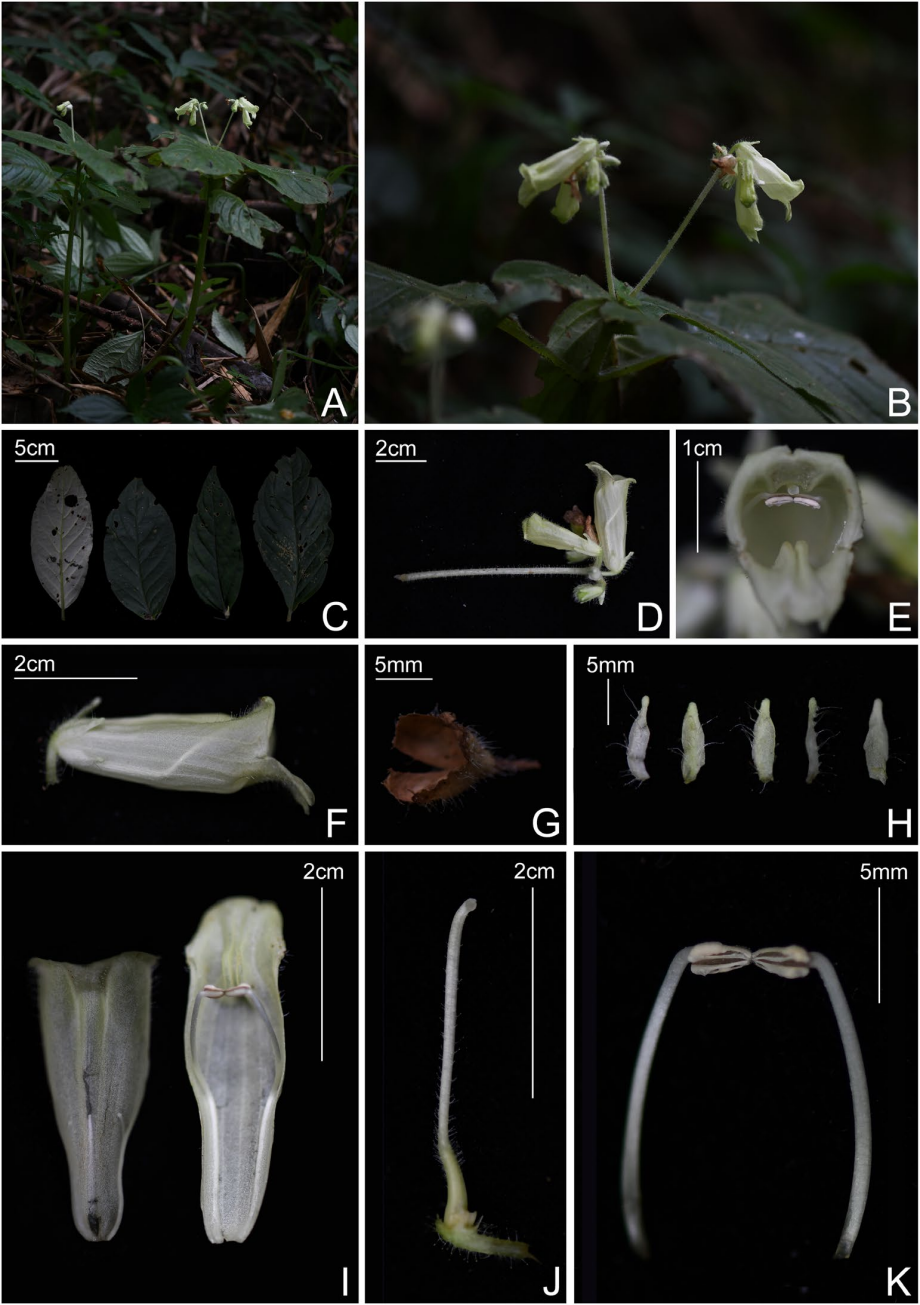
*Hemiboea cehengensis* sp. nov. (A) Habitat; (B) Flowering individual; (C) The adaxial surface of leaf blades and the abaxial surface of leaf blades; (D) Cymes; (E) Front view of corolla; (F) Side view of corolla; (G) Abscission involucre; (H) Opened calyx; (I) Opened corolla; (J) Disc and pistil; (K) Stamens.

Type: CHINA. Guizhou: Ceheng, Wanchongshan Scenic Area, growing in understory shade, alt. 1514.7 m, 24°58′21.37″ N, 105°39′42.2″ E, 21 September 2023, voucher *X.X. Bai Bxx‐bs‐032* (holotype, GZAC!; isotype, GZAC!) (Figure 4).

### 5.10 Diagnosis

The similarities between *Hemiboea cehengensis* and *H. ovalifolia* are as follows: whole plant hairy, involucre early deciduous, two protuberances on the inner abaxial surface of corolla, and absence of a hairy ring at the inner corolla base, but *H. cehengensis* is distinguished by its green stem (vs. brown), absence of purple spots and stripes on the corolla, involucre covered with long glandular hairs (vs. villous), anthers narrowly ovate or broadly ovate (vs. rectangular‐rounded), and sparsely covered with long glandular hairs from the middle upwards of the ovary (vs. densely covered with long glandular hairs from the base of the ovary) (Table 3).

**TABLE 3:** Morphological comparison of *H. cehengensis* and *H. ovalifolia*.

Character	*H. cehengensis*	*H. ovalifolia*
Stem	Green	Brown
Involucre	White, 5–7 mm in diameter, outside densely covered with long glandular hairs	Green, 1.0–2.2 cm in diameter, densely villous
Corolla	No purple spots and stripes	With purple spots or stripes
Anthers	Narrowly ovate or broadly ovate	Rectangular‐rounded
Pistil	Sparsely covered with long glandular hairs above the middle of the ovary	Densely covered with long glandular hairs from the base of the ovary

### 5.11 Description

Perennial herb. Stems 15–60 cm high, simple, subcarnose, densely white pubescent. Leaves opposite; petiole 0.5–5.6 cm long, upper leaves subsessile, densely pubescent. Leaf herbaceous, ovate‐lanceolate, ovate, elliptic or obovate, 9–25 × 6–10 cm, apex acute or acuminate, base slightly oblique, one side cuneate, the other side nearly truncate, margin nearly entire or with inconspicuous undulate teeth, densely white appressed pubescent on both surfaces, lateral veins 7–9 on each side of midrib. Cymes 1–2, subterminal, 10–16 flowered; peduncle 6.1–9.8 cm long, densely white pubescent with long glandular hairs; involucre spheroidal, apex cuspidate, 5–7 mm in diameter, outside densely covered with long glandular hairs, inside glabrous. Pedicel 4.5–9.2 mm long, densely white pubescent with long glandular hairs. Calyx 5‐lobed to base, yellowish green to white, lobes lanceolate‐oblong, apex obtuse, 5.9–9.2 × 1.8–2.2 mm, outside densely covered with long glandular hairs, inside glabrous. Corolla yellow green, 3.5–4.5 cm long, funnelform tube, sparsely glandular‐pubescent outside, inner abaxial surface with two protuberances near the mouth; tube 3.2–4.2 cm long, mouth 1–1.2 cm in diameter; limb 2‐lipped, adaxial lip 2‐lobed, lobes oblique triangle, 2.3–2.9 mm long; abaxial lip 3‐lobed, central lobe ovate to broadly ovate, lateral lobes oblique triangle, 2.1–2.7 mm long. Stamens 2, adnate to 1.5–1.7 cm above the corolla base; filaments linear, 1.2–1.5 cm long, glabrous; anthers narrowly ovate or broadly ovate, 2–3 mm long, apex coherent. Staminodes 3, adnate to 1–1.1 cm above the corolla base, linear, glabrous, central one 1–1.4 mm, lateral two 2–2.9 mm. Disk annular, pale yellow, notched near the middle of the rachis, 1.3–1.7 mm high, margin irregular, erosulate. Pistil 2.2–3.3 cm long; ovary linear, 6.2–8.6 cm long, sparsely covered with long glandular hairs above the middle part; style 1.3–2.4 cm long; densely glandular‐pubescent; stigma truncate. Capsule unknown.

### 5.12 Pollen Description

The pollen grains of *Hemiboea cehengensis* are spherical and rounded in polar view. The pollen grain polar axis length is 18.15–18.23 μm, the equatorial axis length is 16.11–17.61 μm; the germination furrow is 3.20–4.86 μm wide; the outer wall ornamentation is reticulate, with rough ridges, fewer perforations, and rounded and blunt mesh holes whose size and shape are irregular (See Appendix S2 for images).

### 5.13 Phenology

Flowering period: September and October.

### 5.14 Etymology

The etymology of the specific epithet “cehengensis” is Latinized from the Chinese Pinyin of the type locality “Ceheng” with the suffix “ensis”.

### 5.15 Vernacular Name

Simplified Chinese: 册亨半蒴苣苔; Chinese pinyin: cè hēnɡ bàn shuò jù tāi.

### 5.16 Distribution and Ecology

We actively surveyed the surrounding areas of Ceheng County: Anlong County, Zhenfeng County, Wangmo County, Qinglong County, Pu'an County, Xingyi City, and Xingren City, and no distribution points were found for the time being. Only one distribution point of *Hemiboea cehengensis* has been found in Ceheng County, in the scenic area of Wanchongshan Mountain, with 200–300 mature individuals. It grows in the shaded understory of evergreen broad‐leaved forest, and its main companion species are *Chimonobambusa quadrangularis* (Franceschi) Makino and *Whytockia tsiangiana* (Hand.‐Mazz.) A. Weber. This species should be considered Vulnerable (VU), according to IUCN criteria D (IUCN Standards and Petitions Committee 2024).

## Author Contributions

**Ping Liang:** formal analysis (lead), writing – original draft (lead). **Xiao Wang:** conceptualization (equal), formal analysis (supporting), investigation (lead), resources (equal). **Fang Wen:** supervision (equal), writing – review and editing (lead). **Xin‐Xiang Bai:** conceptualization (lead), investigation (equal), resources (lead), writing – original draft (supporting), writing – review and editing (supporting). **Jun Mu:** investigation (equal), resources (equal), supervision (equal).

## Conflicts of Interest

The authors declare no conflicts of interest.

## Supporting information


**Appendix S1:** ece372330‐sup‐0001‐AppendixS1.docx.

**Appendix S2:** ece372330‐sup‐0002‐AppendixS2.docx.

## Data Availability

The DNA sequences generated in the present study have been deposited in the National Center for Biotechnology Information (NCBI) database. The accession numbers and the information on the voucher specimens are available in Table 1. The voucher specimens of the new species were housed in GZAC.
