# Supplementary material for: Phylogenetic Relationships, Character Evolution, and Two New Species in Hemiboea (Didymocarpoideae, Trichosporeae)
Source: Ecol Evol. 2025 Oct 14;15(10):e72330. doi: 10.1002/ece3.72330 (PMC12519500; doi:10.1002/ece3.72330)
Supplement: Supplementary file 1 — Appendix S1: ece372330‐sup‐0001‐AppendixS1.docx. [file ECE3-15-e72330-s001.docx]

Appendix S1.

**
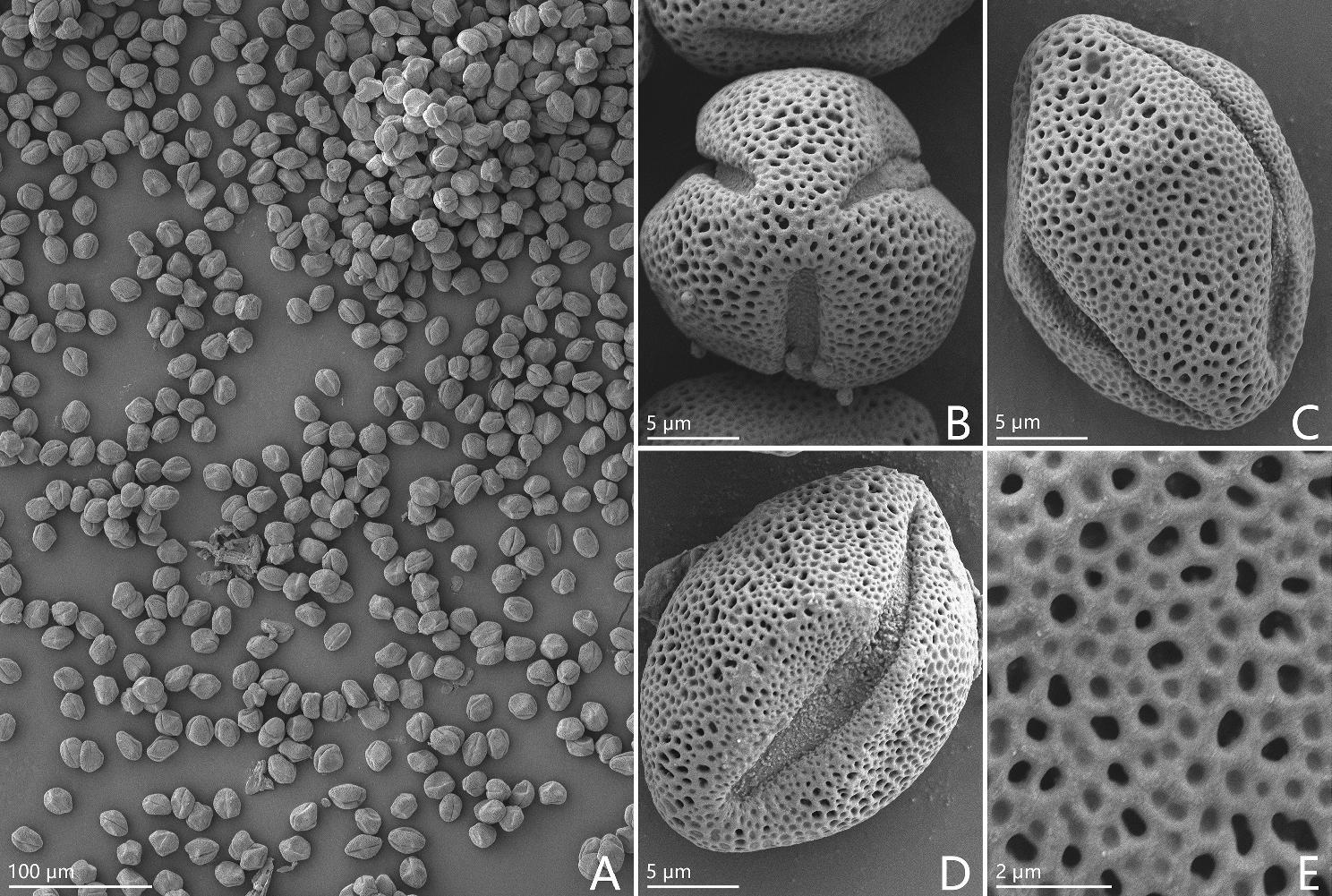
**

Electron microscope scanning pollen morphology of *H. xishuiensis* sp. nov*.* **A**, Ensemble view; **B**, Polar view; **C**, Equatorial view; **D**, Germination grooves; **E**, Pollen wall ornamentation.
